# *PAX1* hypomethylation as a prognostic biomarker for radioresistance of cervical cancer

**DOI:** 10.1186/s13148-023-01538-1

**Published:** 2023-08-02

**Authors:** Xuanxuan Li, Huan Liu, Xue Zhou, Yangying Zhou, Yu Zhang, Yu-Ligh Liou, Manting Zeng, Hong Zhu

**Affiliations:** 1 Department of Oncology, Xiangya Hospital, Central South University, Hunan, 410008 China; 2 Department of Obstetrics and Gynecology, Xiangya Hospital, Central South University, Hunan, 410008 China; 3 Department of Clinical Pharmacology, Xiangya Hospital, Central South University, Hunan, 410008 China

**Keywords:** *PAX1*, Methylation, Cervical cancer, Radioresistance

## Abstract

**Background:**

*PAX1* gene methylation plays an important role in the development of cervical cancer. However, its prognostic value after radiotherapy for locally advanced cervical cancer is unknown, so this study aimed to investigate the value of *PAX1* gene methylation for predicting the sensitivity of radiotherapy for cervical cancer.

**Methods:**

We selected 125 patients with primary cervical cancer who underwent concurrent chemo-radiotherapy as the study population, quantitative methylation-specific polymerase chain reaction (QMSP) was used for detecting *PAX1* methylation status of cervical exfoliated cells. Logistic regression model was used to analyze the risk factors associated with the short-term efficacy and to establish a prediction model of radiotherapy sensitivity based on *PAX1* gene methylation. Cell viability after radiation of Hela and SiHa cells transfected with *PAX1* or control vector was evaluated by CCK8. Furthermore, RNA-Seq analyses identified different expressed genes (DEGs) in *PAX1* overexpressed SiHa cells. Gene Ontology (GO) and pathway enrichment analysis was carried out to determine the biological function of DEGs.

**Results:**

*PAX1* methylation level was associated with HPV16/18-positive rate. *PAX1* hypomethylation was found to be a risk factor for tumor residual after chemo-radiotherapy. A nomogram containing the risk factors for *PAX1* methylation status, lymph node metastasis, pathological type and tumor size was further constructed to predict the probability of tumor residual after chemo-radiotherapy (AUC = 0.823, 95% CI 0.736–0.910). High *PAX1* protein level was more likely to cause radioresistance in both Hela and SiHa cells. Transcriptomic sequencing of *PAX1* overexpressed and control cells identified 615 differentially expressed genes, and GO enrichment analysis suggested that *PAX1* may be involved in the regulation of signaling receptor activity and response to viruses.

**Conclusion:**

*PAX1* hypomethylation status could be used as a promising biomarker to predict radioresistance in cervical cancer. This further provides a new idea for the individualized treatment strategy of simultaneous radiotherapy for cervical cancer.

**Supplementary Information:**

The online version contains supplementary material available at 10.1186/s13148-023-01538-1.

## Background

In China, cervical cancer has always been the most common cancer affecting women’s health [1]. According to the latest report released by the International Cancer Research Center, cervical cancer accounts for approximately 600,000 new cases and 342,000 deaths worldwide each year [2]. Young women in some parts of the world are reportedly at an increased risk of cervical cancer [2].

As one of the main treatment modalities for patients with cervical cancer, radiotherapy can significantly improve cervical cancer prognosis. However, tumor resistance to radiation considerably limits the overall effect of radiotherapy on cervical cancer. Therefore, predicting tumor sensitivity to radiotherapy is beneficial for the stratified management of patients, adjusting treatment methods in time, and reducing side effects. At present, FIGO (International Federation of Gynecology and Obstetrics) staging plays an important role in treatment planning and prognosis evaluation of cervical cancer. However, the 2018 FIGO staging classifies lymph node lesions into the same stage without considering the extent and size of the primary tumor, which can easily lead to a greater difference in survival [3, 4]. Moreover, FIGO staging does not include all the prognostic factors, limiting the accurate judgment of the prognostic risk of patients. Therefore, it is necessary to identify biomarkers for radiotherapy response and establish a predictive model combined with clinical parameters to effectively classify women at a higher risk of radioresistance from those who would benefit from radiotherapy and facilitate targeted treatment.

The abnormal biological behavior of cervical tumors, such as tumor invasion, metastasis, and progression, is closely related to the epigenetic changes of tumor suppressor genes [5, 6]. DNA methylation plays an important role in the epigenetic mechanism that results in the silencing of heritable genes without altering the coding sequence. This can affect almost every step of tumor progression. The abnormal methylation of CpG islands in the promoter region causes the inactivation of tumor suppressor genes. This leads to their reduced expression and even loss of function, inducing the malignant transformation of cells [7].

Many studies previously identified that paired box gene 1 (*PAX1*), a methylation-silenced gene observed in cervical cancer, can be used as an auxiliary biomarker for clinical examination of cervical cancer to improve the effectiveness of screening [8, 9]. *PAX1*, located on the 20p11 chromosome, belongs to a highly conserved family of transcription factors [10]. Many studies have reported the tumor suppressive function of *PAX1* in various types of cancers, such as cervical, oral, and esophageal cancers [8, 11, 12]. Po-Hsuansu et al. [13] have shown that the dynamic balance between intraepithelial kinase and phosphatase is maintained by *PAX1*. *PAX1* suppresses cancer by activating phosphatase via formation of complexes with *SET1B* and *WDR5*, inhibiting the activation of EFG and IL-6 signaling pathways (such as MAPK, SRC, and AKT pathways).

With the continuous in-depth study of the biology of cervical cancer, researchers have discovered a correlation between epigenetic changes including DNA methylation and the effects of radiation on tumor cells [14, 15]. Concurrently, gene methylation changes occur in the early stages of tumor progression before gene mutations and can be detected in noninvasive stroma, such as stool and cervical exfoliated cells. Therefore, the use of gene methylation testing can aid in the early diagnosis of cervical cancer and follow-up of patients after surgery/radiotherapy. The methylation status of another dominant molecule *ZNF582* in cervical cancer screening might predict radiotherapy sensitivity of cervical adenocarcinoma [16]. At present, there is still a lack of research on whether *PAX1* hypermethylation can predict the radiotherapy sensitivity of cervical cancer.

In this study, we detected the status of *PAX1* methylation of tumor exfoliated cells from patients with cervical cancer before concurrent chemo-radiotherapy. We investigated the relationship between *PAX1* methylation and radiotherapy efficiency and established a predictive model based on *PAX1* methylation. In addition, we studied the role of *PAX1* protein to radiosensitivity in vitro and explored the possible mechanism preliminarily.

## Methods

### Study design

Patients with primary cervical cancer who received concurrent chemo-radiotherapy with regular follow-up (once every three months within two years, once every six months between two years and five years, and once a year after five years) at Xiangya Hospital of Central South University between August 2018 and December 2020 were included in this study.

Patients with pathologically diagnosed cervical cancer, complete follow-up data, and no history of surgery, chemotherapy or radiotherapy were included in this study, whereas those with previous treatment history of cervical cancer and cervical lesions, intolerant to radiotherapy, and incomplete follow-up data were excluded. This study was approved by the Ethics Committee of Xiangya Hospital, Central South University (Approval notice: 202010138).

The epidemiological characteristics and clinical data of patients were extracted from medical records, including age, pathological type, FIGO stage (version 2018), anemia, HPV and lymph node metastasis. All patients underwent MRI and gynecological examination for staging and baseline evaluation of tumor size.

### Treatment

All patients included in this study received radiotherapy including external beam radiotherapy and intracavitary brachytherapy. The external beam radiotherapy dose was 1.8–2.0 Gy/d by intensity modulated radiation technique, and the total dose was approximately 45–50 Gy. Intracavitary brachytherapy with a single dose of 6 to 7 Gy was initiated after 15 external irradiations. In addition, patients also received concurrent chemotherapy based on cisplatin during the treatment period.

### Specimen collection, DNA extraction, and DNA methylation determination

Exfoliated cell samples were collected from all patients before radiotherapy. Sample collection was done by an experienced gynecological oncologist. After ensuring that the cervix was completely exposed by vaginal speculum, surface secretions were removed using a cotton swab and exfoliated cells were obtained by swirling a cervical cell sampling brush clockwise 3–5 times on the surface of the cervical tumor. The collected samples were subjected to methylation detection by technicians blinded to the specific clinical information of patients.

DNA from cervical exfoliated cells was extracted using the QIAamp®DNA Mini Kit (Qiagen GmbH, Hilden, Germany) according to the manufacturer’s instructions. The prepared DNA (500 ng) was treated with bisulfite using the EZ bisulfite conversion kit (Zymo Research, Irvine, CA, USA) according to the manufacturer’s instructions. After reacting with sodium bisulfite, unmethylated cytosine is converted to uracil, whereas methylated cytosine remains unaltered. *PAX1* methylation levels were analyzed using TaqMan probe technology and LightCycler® 480 quantitative PCR (qPCR) system (Roche Applied Science, Penzberg, Germany) using the following cycling conditions: incubation at 95 °C for 10 min, 50 cycles of 95 °C for 10 s and 60 °C for 40 s for DNA annealing, and 40 °C for 40 s for extension. Type II collagen gene (*COL2A*) was selected as the internal reference gene to determine whether the amount of DNA in the test sample was sufficient. Each sample could obtain the Cp value of targeted gene and internal control gene. The methylation level was calculated based on the difference between the Cp of the two genes: ΔCp = Cp_target gene_ − Cp_Col2A_ [17]. We define ΔCp ≤ 9 as *PAX1* hypermethylation, otherwise as hypomethylation [18].

### Treatment outcome analysis

The response to radiotherapy was assessed 3 months after conclusion of the entire radiotherapy by MRI and physical examination according to the Response Evaluation Criteria in Solid Tumors (RECIST 1.1) [19]. If no residual tumor was found in gynecological examination and MRI, it was defined as complete response (CR). Partial response (PR) was defined as a reduction of at least 30% in the total diameter of the tumor compared with initial size. If the total diameter of the tumor was observed to increase by at least 20% or if one or more lesions appeared, it was evaluated as a progressive disease (PD). Stable disease (SD) was defined as no sufficient shrinkage to qualify as PR or sufficient increase to qualify as PD.

### Cell culture and plasmid transfection

Human cervical cancer cell lines, HeLa and SiHa, were procured from the American Type Culture Collection (Manassas, VA, USA). Cells were cultivated in RPMI-1640 medium comprising 10% fetal bovine serum in a moist incubator maintained at 37 °C with 5% CO_2_. *PAX1* was overexpressed in HeLa and SiHa cells by tranfecting pCDNA3.1-*PAX1* plasmid obtained from Fenghui Biotechnology (Changsha, China). Transfection was performed in cells via Lipo8000™ Transfection Reagent (#C0533, Beyotime, Nanjing, China). Cells (SiHa and HeLa) were grown in 6-well plates until the cells were about 75% confluent. The growing medium was then changed to 2 ml fresh medium with fetal bovine serum. Plasmid (1.25 µg) and lipo8000 (4 µl) mixed in 125ul RPMI-1640 medium were added slowly into each well. Cells were then cultured 48 h to verify transfection efficiency or subsequent irradiation. Transfection efficiency was confirmed by reverse transcription‑ qPCR and western blot.

### CCK8 assay

HeLa and SiHa cells with vector transfection were seeded in 96-well plates at a density of 3 × 10^3^ per well. The cells were irradiated when they were well adhered to the bottom of plates. It was about 48 h after plasmid transfection. After 6 Gy irradiation, cells were cultured for 48 h. CCK8 reagent was subsequently added in each well, and cells were incubated at 37 °C for 1 h. The optical density (OD) was measured at 450 nm using a microplate reader to calculate the relative viable cells.

### Transcriptome sequencing and enrichment analysis

Total RNA was extracted from SiHa cells transfected with *PAX1* or control vector. RNA integrity was assessed using the RNA Nano 6000 Assay Kit of the Bioanalyzer 2100 system (Agilent Technologies, CA, USA). Samples were sent to Novogene Corporation (Shanghai, China) for further RNA-seq detection and analysis. The clustering of the index-coded samples was performed on a cBot Cluster Generation System, and then the library preparations were sequenced on an Illumina Novaseq platform. Differential expression analysis of two samples was performed using the edgeR R package (3.22.5). *P*-values were adjusted using the Benjamini–Hochberg method. Corrected *P*-value of 0.05 and absolute foldchange of 2 were set as the threshold for differentially expressed genes (DEGs). Enrichment analysis of DEGs including Gene Ontology (GO) enrichment, KEGG pathway and Reactome pathway analysis was performed. Enrichment analysis was executed by clusterProfiler software (3.4.4). GO terms or pathways with corrected *P*-value < 0.05 were considered significantly enriched.

### Statistical analysis

The acquired data were statistically analyzed using SPSS25.0 and R language statistical software packages and visualized using Graphpad Prism (8.0.1). All quantized values in normal distribution are expressed as mean ± standard deviation, and counting data are expressed by constituent ratio or percentage. Student’s *t* test was performed for data in line with normal distribution, and Mann–Whitney *U* test was performed for data that did not conform to normal distribution. All tests were conducted with bilateral 95% confidence interval (CI), and the difference was considered to be statistically significant.

Differences in clinical parameters and *PAX1* methylation status between different treatment groups were evaluated by independent sample *t*-test, *χ*^2^ test, or Fisher’s exact test. To establish an individualized line chart model comprising independent risk factors to predict the risk of tumor residue, the impact of the following parameters on radiotherapy response was evaluated: patient’s age, FIGO stage, hemoglobin levels, HPV, tumor size, and *PAX1* methylation. Regression logistics were used for univariate and multivariate analyses. In univariate analysis, the variables with *P*-value < 0.05 entered the covariates of multivariate models, and the odds ratios (ORs) and CIs of the independent variables were calculated. Independent risk factors achieving statistical significance in the multivariable logistic regression model were introduced into RStudio. An individualized nomogram to predict the short-term efficacy was established using package RMS. Bootstrap self-sampling method was used to verify the model internally. The discrimination and consistency of logistics regression models was assessed using the receiver-operating characteristic curve (ROC) and the calibration plot. Hosmer–Lemeshow (goodness of fit) test was used to evaluate the fitting degree of the scoring model.

## Results

### *PAX1* gene methylation is associated with HPV16/18

In this study, we analyzed data from 125 patients with a median age of 56 years and average age 56.0 ± 9.0 years. The baseline clinical characteristics and tumor molecular feature of patients in cervical cancer are summarized in Table 1.

**Table 1:** Patients characteristics and the correlation between clinical variables and *PAX1* gene methylation

Variables	*N* = 125	Hypomethylation (*N* = 15)	Hypermethylation (*N* = 110)	*P*
Age (year)	56.0 ± 9.0	57.8 ± 6.6	55.8 ± 9.2	0.415^a^
*FIGO stage*				0.761^b^
< IIB	10	2 (20.0%)	8 (80.0%)	
≥ IIB	115	13 (11.3%)	102 (88.7%)	
*Pathology*				0.152^c^
SCC	119	13 (10.9%)	106 (89.1%)	
AC	6	2 (33.3%)	4 (66.7%)	
*Lymph nodes*				0.326^d^
Negative	73	7 (9.6%)	66 (90.4%)	
Positive	52	8 (15.4%)	44 (84.6%)	
*HPV16/18*				0.049^*^^d^
Negative	39	8 (20.5%)	31 (79.5%)	
Positive	86	7 (8.1%)	79 (91.9%)	
*Anemia*				0.819^d^
Yes	45	5 (11.1%)	40 (88.9%)	
No	80	10 (12.5%)	70 (87.5%)	
Tumor size (cm^3^)		19.0 ± 24.1	23.6 ± 25.3	0.508^a^

HPV, Human papillomavirus; FIGO, International Federation of Gynecology and Obstetrics; SCC, squamous cell carcinoma; AC, adenocarcinoma

^a^*t* test

^b^Continuous correction *χ*^2^ test

^c^Fisher test

^d^*χ*^2^ test

^*^Statistical significance

First, to investigate whether the *PAX1* methylation status was correlated with clinical variables of cervical cancer, 125 patients were categorized into two groups according to the interpretation criteria of ΔCp value: *PAX1* hypermethylated group (n = 110, 88%) and *PAX1* hypomethylated group(n = 15, 12%). Additional file 1: Fig. 1 shows the distribution of ΔCp value for hypermethylation and hypomethylation. The initial *PAX1* methylation status showed a positively correlation with HPV16/18. The results indicate that HPV16/18 status may affect PAX1 methylation status. No significant differences in age, FIGO stage, pathological type, tumor size, lymph node status, anemia were observed between subjects with hypomethylated *PAX1* and hypermethylated *PAX1*.

### Independent prognostic factor in radiation response of patients with cervical cancer

After treatment outcome analysis, 125 patients were divided into two groups: CR group comprising 90 patients and residual disease group (PR + PD + SD) comprising 35 patients. Univariate and multivariate logistics regression analyses were performed to determine whether *PAX1* methylation is an independent risk factor for poor prognosis in cervical cancer.

According to the univariate binary logistics regression analysis, tumor size (*p* = 0.000), *PAX1* status (*p* = 0.026), and lymph node metastasis (*p* = 0.000) were associated with sensitivity to radiotherapy. The multivariate logistics analysis identified that tumor size [1.038 (1.014–1.062)], *PAX1* hypomethylation [4.433 (1.131–17.380)], lymph node metastasis [2.854 (1.067–7.638)], and pathological type [7.473 (1.011–55.254)] were independent risk factors for residual tumor after radiotherapy in cervical cancer (Table 2). The results showed that hypomethylated *PAX1* positively correlated with residual tumor after radiotherapy, indicating that the radiosensitivity of patients with hypomethylated *PAX1* was lower than those with hypermethylated *PAX1*.

**Table 2:** Univariate and multivariate logistic regression analysis of radiosensitivity of cervical cancer

Characteristics	Univariate analysis				Multivariate analysis			
	*β*	OR	95% CI	*P*	*β*	OR	95% CI	*P*
Age (year)	− 0.007	0.993	0.951–1.038	0.757				
FIGO stage	0.476	1.610	0.325–7.984	0.560				
Pathology	1.736	5.677	0.990–32.542	0.051	2.011	7.473	1.011–55.254	0.049*
Lymph nodes	1.764	5.833	2.466–13.797	< 0.001*	1.049	2.854	1.067–7.638	0.037*
HPV16/18	− 0.015	0.985	0.425–2.286	0.973				
Anemia	0.405	1.500	0.674–3.339	0.321				
Tumor size	0.039	1.040	1.019–1.062	< 0.001*	0.037	1.038	1.014–1.062	0.002*
*PAX1* hypomethylation	1.257	3.513	1.165–10.590	0.026*	1.489	4.433	1.131–17.380	0.033*

CI, Confidence interval; OR, odds ratio; HPV, human papillomavirus; FIGO, International Federation of Gynecology and Obstetrics; SCC, squamous cell carcinoma; AC, adenocarcinoma

^*^Statistical significance

### Development and validation of nomogram based on *PAX1* methylation

**Fig. 1:**
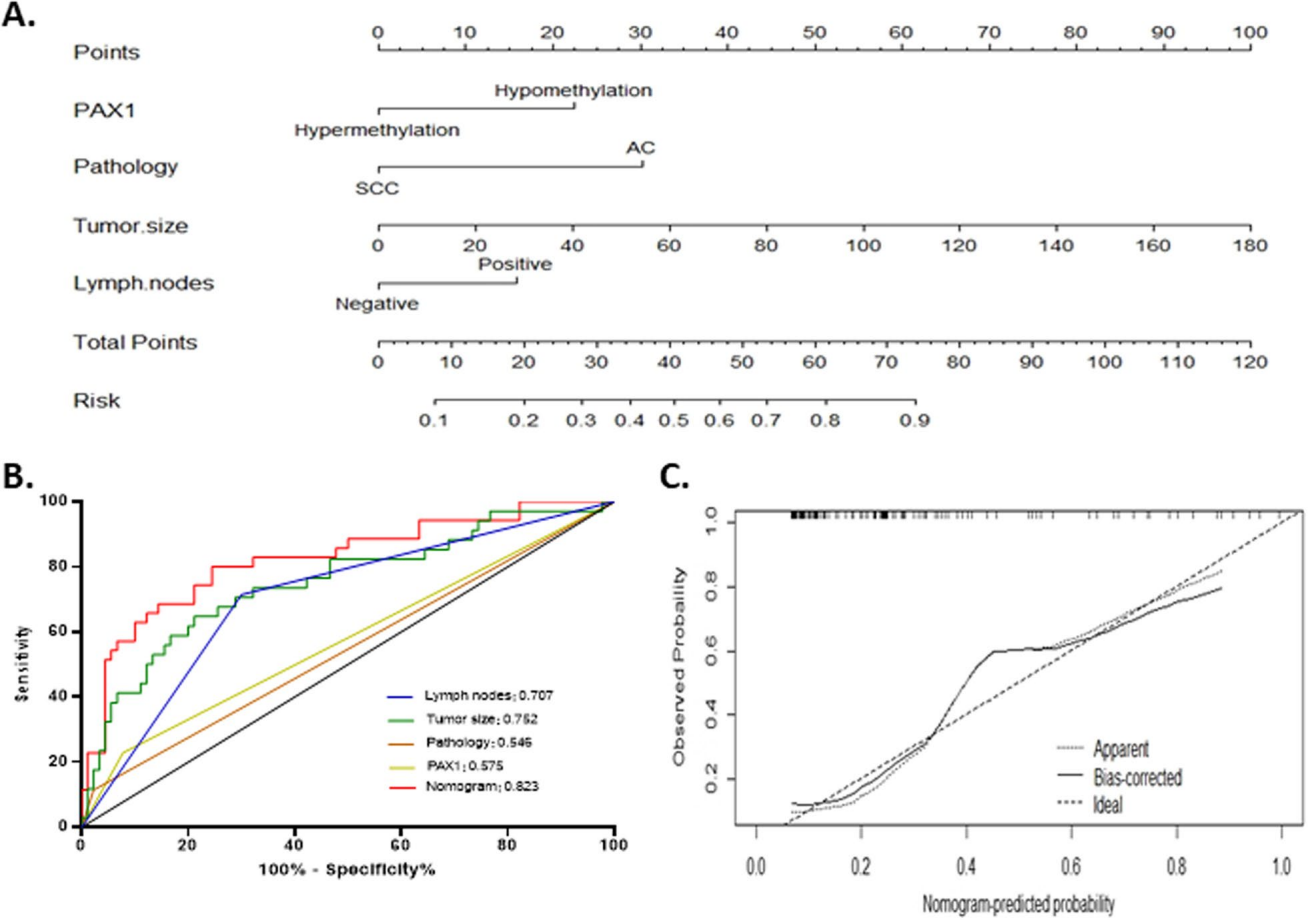
**A** Nomogram for predicting tumor residual risk in cervical cancer underwent radiotherapy. **B** Receiver operating characteristic curve (ROC) of nomogram and variables for prediction residual tumor in cervical cancer. **C** Calibration plot of the predicted and observed probabilities of residual tumor. SCC, squamous cell carcinoma; AC, adenocarcinoma

Through multivariate logistics regression analysis, a nomogram based on *PAX1* methylation for predicting the radiotherapy sensitivity of patients with cervical cancer was constructed by R software (Fig. 1A).

According to the nomogram, the corresponding score of each predictive variable can be obtained at the top scale, and the predictive risk corresponding to the sum of score represents the residual tumor risk. For example, in our study, the MRI of a 57-year-old patient who presented with adenocarcinoma revealed primary tumor size of 8 cm^3^, lymph node negative, and FIGO stage IIA1. The *PAX1* methylation status at pretreatment was hypomethylation. Using the nomogram, we predicted that the corresponding residual risk after radiotherapy was 0.747 (Additional file 1: Fig. 2). And we found in our clinical follow-up that this 57-year-old patient did have a residual mass after radiotherapy and had distant metastases 1 year after treatment.

The gene-based risk score prognostic predictor was then tested in the internal validation. Validation of the nomogram was performed with 1000 bootstrapped samples. The discriminant ability of nomogram and each single factor variable was evaluated using ROC curve. Area under the ROC curve (AUC) demonstrated the ability of our nomogram model in predicting radiotherapy sensitivity (Fig. 1B). The results showed that AUC of model is 0.823 (95% CI 0.736–0.910), whereas the sensitivity and specificity at the best intercept are 80.00% and 75.56%, respectively. The result of H–L goodness of fit test (*χ*^2^ = 4.768, *P* = 0.782 > 0.05) showed that the model has a good fit. The calibration curve also showed that the bias-corrected line of nomogram model was close to ideal curve, which indicated a good agreement between predicted value and actual observed value (Fig. 1C).

### *PAX1* protein expression is associated with radioresistance in cervical cancer

**Fig. 2:**
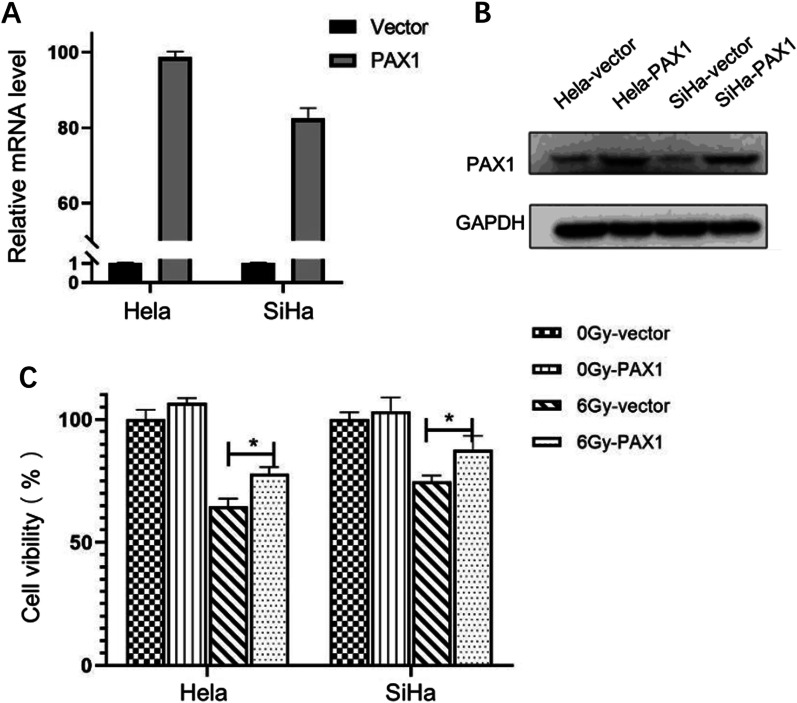
**A** and **B** *PAX1* mRNA and protein level in Hela and SiHa cells when cells were transfected with control vector and *PAX1* vector. **C** Cell viabilities of HeLa and SiHa cells after 6 Gy radiation treatment for 48 h

Furthermore, we tested whether PAX1 could induce radioresistance in cervical cancer cell lines. We induced *PAX1* overexpression in SiHa and HeLa cells by vector transfection. *PAX1* mRNA and protein levels were confirmed by qRT-PCR and Western blot, respectively (Fig. 2B, C). After irradiating cells for 48 h, CCK8 assay showed that cells overexpressing *PAX1* had higher survival rates compared with control cells.

### *PAX1* overexpression in SiHa cells cause transcriptome difference

**Fig. 3:**
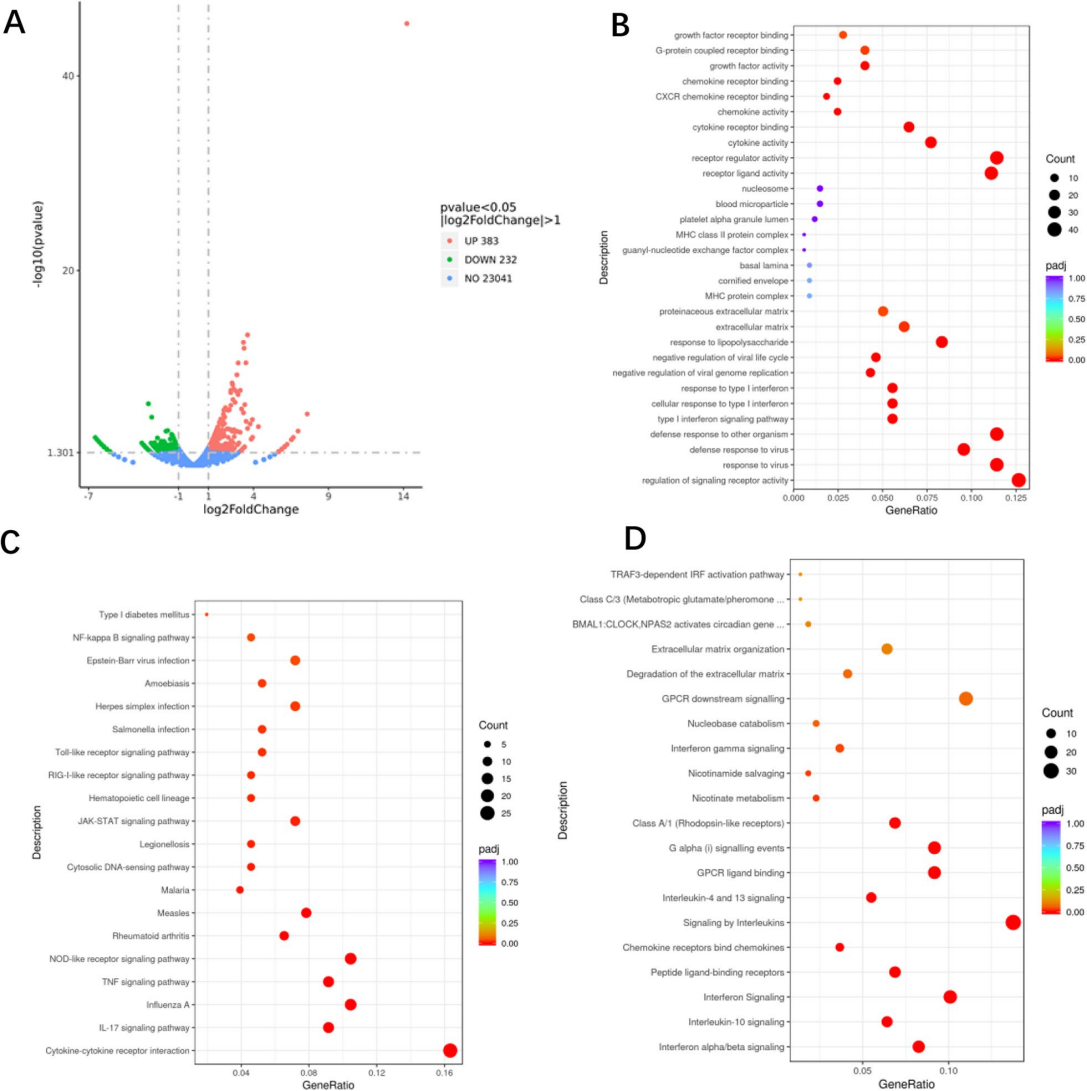
**A** Volcano of differential expression genes in SiHa cells transfected with *PAX1* vector or control vector. **B** Gene Ontology (GO) enrichment analysis. **C** KEGG enrichment analysis. **D** Reactome enrichment analysis

We studied the potential mechanism of *PAX1* causing radioresistance in cervical cancer cells by transcriptome analysis. Compared to control vector, paired differential gene expression analysis revealed 615 genes differentially expressed. Among the DEGs, 383 and 232 genes were upregulated and downregulated, respectively, in *PAX1*-overexpressing SiHa cells (Additional file 2). A volcano plot of DEGs is presented in Fig. 3A.

Through GO analysis of DEGs, we found that the genes clustered in 185 significant GO categories, including regulation of signaling receptor activity and response to virus; the main categories are presented in Fig. 3B. KEGG pathway enrichment analysis identified 21 significant pathways related with the DEGs, such as cytokine-cytokine receptor interaction (Fig. 3C). Reactome enrichment revealed 14 significant reactions and biological pathways, including interferon alpha/beta signaling (Fig. 3D).

## Discussion

Radioresistance due to tumor heterogeneity, biological behavior, and other factors limits the efficacy of radiotherapy in some patients [20]. Identifying patients at highest risk of residual tumor prior to radiotherapy initiation can provide an opportunity for modifying therapy. In previous studies, most predictors of radiotherapy sensitivity in cervical cancer were clinicopathological parameters, and there is a lack of commonly accepted effective biomarkers. Our study illustrated that hypomethylated *PAX1* before treatment was significantly related to unfavorable short-term radiotherapy efficacy. We also combined *PAX1* methylation with clinical variables to establish a visual prediction model. *PAX1* methylation detection is accessible, noninvasive, and safe. Thus, *PAX1* gene methylation is a promising biomarker of radiosensitivity of cervical cancer.

In recent years, gene promoter methylation studies have provided more evidence for the diagnosis, radiation resistance, clinical prognosis, and monitoring of malignant carcinoma [14]. With the increasing focus on epigenetics, *PAX1* methylation has also attracted the attention of researchers. Clinically, most *PAX1* methylation studies initially focused on the screening of cervical cancer [17]. These studies demonstrated that *PAX1* gene is silenced by promoter methylation in cervical cancer and proposed it as a molecular biomarker for cervical cancer screening and diagnosis; however, it has been barely studied for radiation response in cervical cancer.

Our results revealed that radiotherapy response of patients with cervical cancer could be attributed to *PAX1* methylation status; hypomethylated *PAX1* correlated with an increasing incidence of radiation resistance in patients with cervical cancer. In this study, *PAX1* hypomethylation, tumor size, pathological type, and lymph node status were identified as independent risk factors for radiotherapy response.

LimA et al. [21] in a retrospective study demonstrated that lymph node metastasis led to a 6.25-fold increased risk of disease recurrence, a 5.15-fold increased risk of death, and a 35–40% decrease in overall survival. Joanna Jonska-Gmyrek et al. [22] found that patients with adenocarcinoma were more likely to die from disease (HR: 1.60; 95% CI 1.26–2.58) and disease recurrence (HR: 1.69; 95% CI 1.21–2.12). Also tumor volume was considered to be one of the factor affecting the efficacy of radiotherapy for patients with locally advanced cervical cancer. In this study, FIGO stage was not an independent predictor of whether tumors remained after concurrent chemo-radiotherapy for cervical cancer probably because the study population predominantly included intermediate to advanced stage patients. Therefore, we believe that other clinical factors should also be considered in the selection of treatment options.

Subsequently, these predictors were incorporated into a predictive nomogram to calculate the risk probability of tumor residuals tailored to individual patients. We further found that the nomogram had good predictive performance for radiotherapy response with AUC of 0.823, and the sensitivity and specificity under the optimal cutoff value were 80.00% and 75.56%, respectively. Additionally, calibration curves showed that the deviation correction curve of the prediction model established in this study was basically consistent with the ideal curve. In our study, *PAX1* methylation was introduced as a new parameter to create a better model for calculating the probability of local residuals after radiotherapy for each patient. Epigenetic-based nomograms can provide a more comprehensive and accurate prediction of disease prognosis than FIGO staging and can also be used as a means of patient stratification. The ability to identify patients with the highest residual risk prior to treatment can provide an opportunity to adjust therapy.

To clarify the mechanism of *PAX1* methylation involved in radioresistance in cervical cancer, we overexpressed *PAX1* gene in cervical cancer cell lines, and we found that cervical cancer cells overexpressing *PAX1* had a higher survival rate after 6 Gy of radiation exposure. It is well known that persistent high-risk HPV16/18 infection is the main cause of cervical cancer [23]. It has been reported that E6 and E7 are transforming genes of HPV16 that independently induce malignant transformation [24]. HPV16 E6 may confer an aggressive radiation-resistant phenotype [25]. Multiple studies have found that HPV can promote radioresistance through many possible pathways, like inducing CD71, CD55, and Nurr1 [26–28]. In this study, *PAX1* methylation was clinically relevant to the HPV16/18 infection, with higher levels of *PAX1* methylation in HPV16/18-positive patients than in HPV16/18-negative patients. However, our univariate analysis showed that HPV16/18 positivity or negativity did not affect the efficacy of radiotherapy in patients with cervical cancer. We propose that the main role of the presence of HPV16/18 is to induce *PAX1* promoter methylation in host cells, and *PAX1* methylation further proceeds to affect radiotherapy efficacy. It has been reported that HPV is the activator of DNA methyltransferase [29]. Both E6 and E7 oncoproteins encoded by HPV16 affect the direct binding of DNA methyltransferase DNMT1 E7 protein to DNMT1 and stimulate DNA methyltransferase activity [29]. *PAX1* methylation level is higher in HPV16/18-positive patients than in HPV16/18-negative patients. RNA-seq analysis shows GO enrichment items of DEGs, including response to virus, defense response to virus, and negative regulation of viral genome replication/viral life cycle. KEGG enrichment also identified NOD-like receptor signaling pathway, which regulates antiviral innate immune response. We speculate that *PAX1* may also play a synergistic role in the process of cervical carcinogenesis and radioresistance induced by HPV16/18.

Based on our previous study [30], we speculated that *PAX1* shares a certain regulatory relationship between genes and ionizing radiation. The loss of *PAX1* function caused by *PAX1* methylation may affect the immune function, whereas demethylation caused by radiotherapy reactivates the normal regulatory function of *PAX1* on the immune system, causing changes in tumor microenvironment and affecting radiosensitivity. Furthermore, *PAX1* plays an important role in the development of thymus [31]. In this study, RNA-seq data identified some differentially expressed immunoregulatory factors, like chemokines and interleukin family members. KEGG pathway enrichment analysis revealed many signaling pathways, such as IL-17 signaling pathway, Toll-like receptor signaling pathway, and RIG-I-like receptor signaling pathway, involved in immune regulation. *PAX1* might regulate immune system, causing changes in tumor microenvironment and affecting radiosensitivity.

In summary, *PAX1* gene methylation status was a potential prognostic biomarker, our study innovatively studied the role of *PAX1* in radioresistance and established a visual nomogram to predict the risk of residual tumor in patients with cervical cancer receiving radiotherapy. Nevertheless, the present study also has some limitations. First, the sample size of this study was relatively small. Second, the prediction model lacks external verification. Lastly, as the underlying mechanism of *PAX1* inducing radioresistance remains unclear, more studies are required for further verification.

## Conclusion

The present study demonstrated that *PAX1* hypomethylation could be used as a promising biomarker to predict radioresistance in cervical cancer. This further provides a new idea for the individualized treatment strategy of simultaneous radiotherapy for cervical cancer.

## Supplementary Information

**Additional file 1: Figure S1** ΔCp value distribution of PAX1 hypermethylation and hypomethylation. **Figure S2** Example of a nomogram based on *PAX1* gene methylation

**Additional file 2:** Transcriptome sequencing and enrichment analysis data.

## Data Availability

All data generated are included in this article and its Additional files 1 and 2.

## Abbreviations

FIGO: International Federation of Gynecology and Obstetrics
*PAX1*: Paired box gene 1
HPV: Human papillomavirus
MRI: Magnetic resonance imaging
RECIST: Response evaluation criteria in solid tumors
GO: Gene ontology
DEGs: Differentially expressed genes
ROC: Receiver operating characteristic curve
AUC: Area under the curve
QMSP: Quantitative methylation-specific polymerase chain reaction
SCC: Squamous cell carcinoma
AC: Adenocarcinoma
