# Supplementary material for: PAX1 hypomethylation as a prognostic biomarker for radioresistance of cervical cancer
Source: Clin Epigenetics. 2023 Aug 2;15:123. doi: 10.1186/s13148-023-01538-1 (PMC10398938; doi:10.1186/s13148-023-01538-1)
Supplement: Supplementary file 1 — Additional file 1: Figure S1 ΔCp value distribution of PAX1 hypermethylation and hypomethylation. Figure S2 Example of a nomogram based on PAX1 gene methylation [file 13148_2023_1538_MOESM1_ESM.docx]

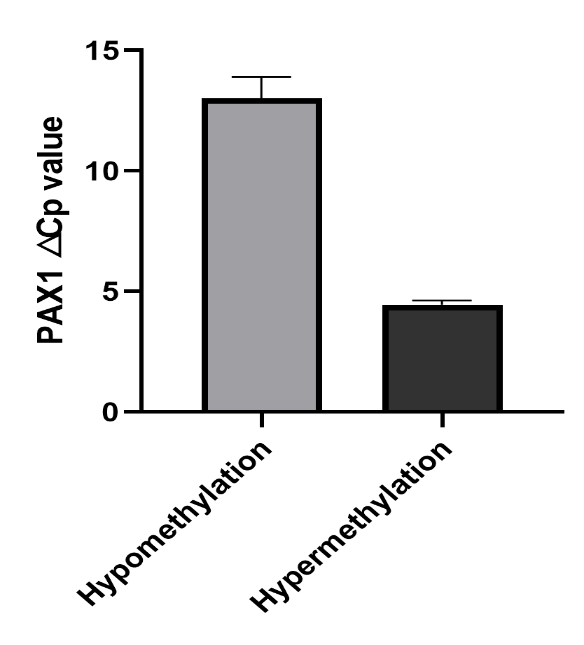


**Supplementary Fig1** △Cp value distribution of *PAX1* hypermethylation and hypomethylation


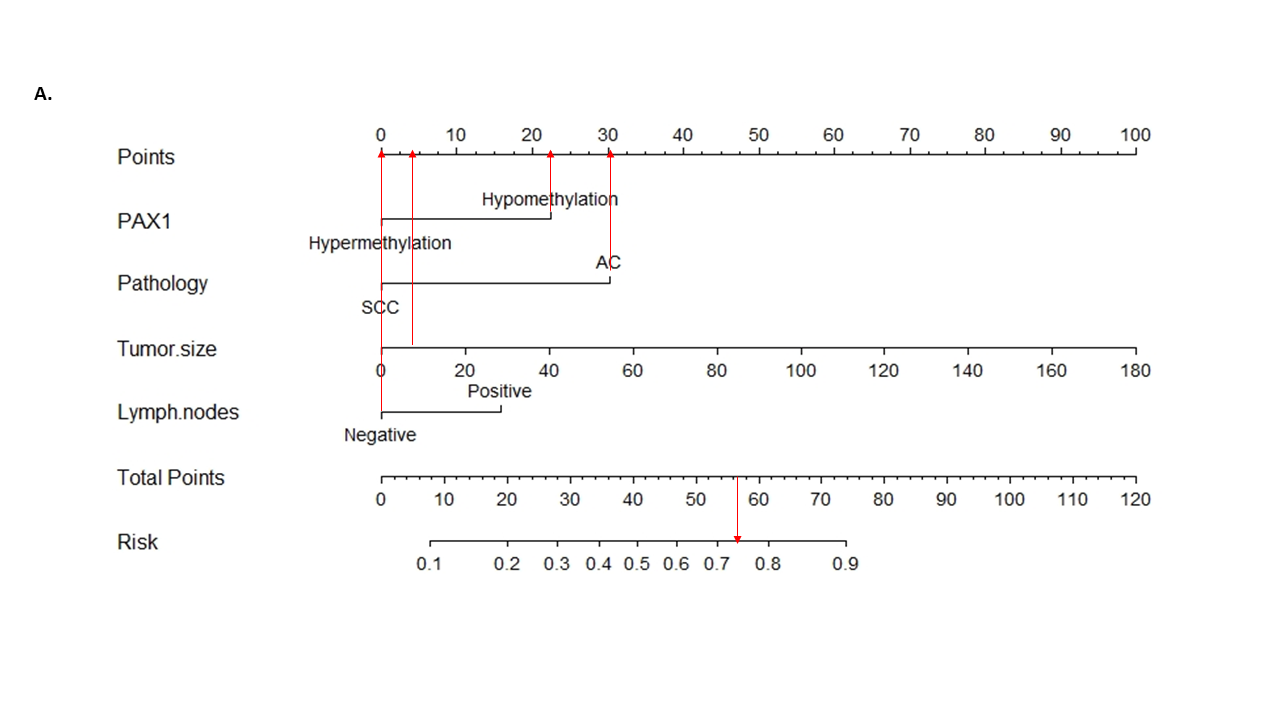


**Supplementary Fig2** Example of a nomogram based on *PAX1* gene methylation
